# Supplementary material for: Quality assessment of endotoxin contamination in consumables used for assisted reproductive technology
Source: PLoS One. 2026 Jun 17;21(6):e0341246. doi: 10.1371/journal.pone.0341246 (PMC13274850; doi:10.1371/journal.pone.0341246)
Supplement: Attachment — Submitted filename: Response to Reviewers.docx [file pone.0341246.s002.docx]

Reply to comments of reviewers and journal requirements

Manuscript number: PONE-D-25-68905

Title: Quality assessment of endotoxin contamination in consumables used for assisted reproductive technology

Authors: Hiroyuki Tomari, et al.

We sincerely appreciate your comprehensive and insightful review of our manuscript. Your constructive comments have been invaluable in helping us improve the quality and clarity of our work. We have responded to your comments below and made the necessary revisions accordingly. All corrections are listed in the attached “List of Changes”.

**Reviewer 1: comment 1**

The study suggests that contaminated containers “may reduce ART success rates,” but no clinical outcome data are provided to support this claim. Extrapolating from laboratory results (embryo growth and sperm motility) of potential biological effects to actual clinical outcomes of ART is not conclusive. Therefore, conclusions about reduced ART success rates should be interpreted in the discussion section appropriately, with a review of previous research, or supported by clinical outcome data.

【Reply】 Deletion and modification

We completely agree with this comment. Since the impact of pyrogen contamination levels identified by HCPT on ART success rates is discussed in the “Discussion” section (the revised manuscript; page 18, lines 381 to 387) with citations to the relevant literature reported to date, we deleted the first sentence and modified the second sentence in the 'Conclusion' section as they were not appropriate for the conclusion of our manuscript. For details of the revised text, please refer to Section 4 in the attached “List of Changes”.

**Reviewer 1: comment 2**

In this study, the observed concentrations of hydrogen peroxide were compared with the reported toxicity threshold for human mesenchymal stem cells. However, mesenchymal stem cells are not biologically equivalent to human gametes or embryos, which are generally known to be more sensitive to oxidative stress. Therefore, using MSC data as a reference for reproductive cell safety may not be appropriate. This section is discussed in detail in the Discussion.

【Reply】 Deletion, modification, and addition

According to this comment, the discussion comparing the effects of hydrogen peroxide on hMSCs and germ cells was deleted and reconstructed by using several references evaluating the effects of hydrogen peroxide on germ cells. The discussion of the results obtained in this study was also revised to ensure consistency with the context. With this revision, new references 36-40 were added. For details of the revised text, please refer to Sections 3/5), 3/6), and 5/1) in the attached “List of Changes”.

**Reviewer 1: comment 3**

The human cell-based pyrogen test (HCPT) plays a key role in the conclusions of this study; more details on the validation and robustness of this method would be helpful.

【Reply】 Addition

Although the characteristics of HCPT and the status of its standardization in the United States, Europe, and Japan are originally summarized in the ‘Discussion’ section (revised manuscript; page 17, lines 369 to 375), further information useful to readers was added in response to Comments 3-5 from Reviewer 1. With the revision, new reference 32 was added, and the original references after no.32 were renumbered. For details of the revised text, please refer to Sections 3/1) and 5 in the attached “List of Changes”.

**Reviewer 1: comment 4**

Were spike and recovery tests performed for the specific substances tested?

【Reply】 Addition

Although spike-and-recovery tests of the cell culture utensils and medical devices used in this study were not performed, such tests have been conducted in our laboratory and other research teams using many products consisting of plastics and metals. Compared with standard endotoxin tests using water-for-injection extracts as samples, the direct HCPT offers up to 2,000-fold, 80-fold, and 800-fold higher sensitivity for detecting pyrogens from metal, plastic, and rubber materials, respectively.

The difference in recovery ratios between the HCPT and endotoxin tests are already mentioned in the 'Introduction' and 'Discussion' sections. However, based on the comments 3 and 4, new reference 32 was added to the “Discussion” section as a representative example of a recovery test (see reply to comment 3).

**Reviewer 1: comment 5**

Was inter-donor variability assessed? How many independent blood donors were included in each test condition?

【Reply】 Addition

Two blood donors participated in this study, and HCPT was performed several times on different dates. As pointed out in this comment, responsiveness to pyrogens in the HCPT varies not only among donor but also according to the blood collection date, even the same donor. Therefore, responsiveness was corrected using IL-6/standard endotoxin and absorbance/standard endotoxin calibration curves generated for each test, enabling accurate quantification of pyrogens. This information was added to the ‘Discussion’ section based on comments 3 and 5 described above (see reply to comment 3).

**Reviewer 2: comment 1**

This work is relevant and should be published. Small linguistic issues, however: Headers: "Effect on mice and human embryo development" should be "Effect on mouse and human embryo development"; "Removal performance of residual hydrogen peroxide by drying treatment" should be "Removal of residual hydrogen peroxide".

【Reply】 Modification

The headers in the ‘Results’ section were modified according to this comment.

**Academic editor: Journal requirement 1**

Please ensure that your manuscript meets PLOS ONE's style requirements, including those for file naming. The PLOS ONE style templates can be found at https://journals.plos.org/plosone/s/file?id= wjVg/ PLOSOne_formatting_sample_main_body.pdf and https://journals.plos.org/plosone/s/file?id= ba62/ PLOSOne _ formatting_sample_title_authors_affiliations.pdf.

【Reply】 It has been confirmed before submission of the revised version.

**Academic editor: Journal requirement 2**

We note that your Data Availability Statement is currently as follows: “All relevant data are within the manuscript and its Supporting Information files.”

Please confirm at this time whether or not your submission contains all raw data required to replicate the results of your study. Authors must share the “minimal data set” for their submission. PLOS defines the minimal data set to consist of the data required to replicate all study findings reported in the article, as well as related metadata and methods (https://journals.plos.org/plosone/s/data-availability#loc-minimal-data-set-definition).

For example, authors should submit the following data:

- The values behind the means, standard deviations and other measures reported;

- The values used to build graphs;

- The points extracted from images for analysis.

Authors do not need to submit their entire data set if only a portion of the data was used in the reported study.

If your submission does not contain these data, please either upload them as Supporting Information files or deposit them to a stable, public repository and provide us with the relevant URLs, DOIs, or accession numbers. For a list of recommended repositories, please see https://journals.plos.org/ plosone/s/recommended-repositories.

If there are ethical or legal restrictions on sharing a de-identified data set, please explain them in detail (e.g., data contain potentially sensitive information, data are owned by a third-party organization, etc.) and who has imposed them (e.g., an ethics committee). Please also provide contact information for a data access committee, ethics committee, or other institutional body to which data requests may be sent. If data are owned by a third party, please indicate how others may request data access.

【Reply】 No Changes

All relevant data are included in the manuscript.

**Academic editor: Journal requirement 3**

Thank you for stating the following in the Financial Disclosure section: “The author(s) received no specific funding for this work.” We note that one or more of the authors are employed by a commercial company: “Innovative Decontamination Technology Development and Evaluation Center, MIURA CO. LTD., Yokohama-shi, Kanagawa, Japan” and “Low-Temperature Sterilizer Technical Section, Medical Hospital Equipment Technical Department, MIURA CO. LTD., Matsuyama-shi, Ehime, Japan”

1. Please provide an amended Funding Statement declaring this commercial affiliation, as well as a statement regarding the Role of Funders in your study. If the funding organization did not play a role in the study design, data collection and analysis, decision to publish, or preparation of the manuscript and only provided financial support in the form of authors' salaries and/or research materials, please review your statements relating to the author contributions, and ensure you have specifically and accurately indicated the role(s) that these authors had in your study. You can update author roles in the Author Contributions section of the online submission form.

Please also include the following statement within your amended Funding Statement. “The funder provided support in the form of salaries for authors [insert relevant initials], but did not have any additional role in the study design, data collection and analysis, decision to publish, or preparation of the manuscript. The specific roles of these authors are articulated in the ‘author contributions’ section.” If your commercial affiliation did play a role in your study, please state and explain this role within your updated Funding Statement.

2. Please also provide an updated Competing Interests Statement declaring this commercial affiliation along with any other relevant declarations relating to employment, consultancy, patents, products in development, or marketed products, etc. Within your Competing Interests Statement, please confirm that this commercial affiliation does not alter your adherence to all PLOS ONE policies on sharing data and materials by including the following statement: "This does not alter our adherence to  PLOS ONE policies on sharing data and materials.” (as detailed online in our guide for authors http://journals.plos.org/ plosone/s/competing-interests) . If this adherence statement is not accurate and there are restrictions on sharing of data and/or materials, please state these. Please note that we cannot proceed with consideration of your article until this information has been declared.

Please include both an updated Funding Statement and Competing Interests Statement in your cover letter. We will change the online submission form on your behalf.

【Reply】 Modification

We understand that if any of the authors are affiliated with commercial entities, it will be subject to rigorous scrutiny regarding potential conflicts of interest. For this reason, not only for PLOS One but for all journal submissions, we have provided the ICMJE Disclosure Form for all authors as ‘Supplementary Information’ to assist the Chief Editor in making a decision.

This study did not receive any specific grant from funding agencies in the public, commercial, or not-for-profit sectors. MIURA CO.,LTD. has not provided any research funding to IVF Nagata Clinic, a collaborative research partner, and each facilities conducted the research using its own in-house budgets. Including patent application status related to this study, all information are disclosed in the ICMJE Disclosure Forms. In addition, regardless of the type of organization, fair salaries and in-house budgets are generally not subject to disclosure under the Transparency Guidelines. From these reasons, we previously inquired via email about the necessity of this requirement, but we have not received a response to date.

Since we have never been asked to provide such additional statements from scientific journal offices in past, we understood this to be a specific editorial policy of PLOS One. Although the content seems to be the same and just expressed differently, the Funding Statement and Competing Interests Statement have been updated as follows and also included in the cover letter submitted with our revised manuscript, as requested.

**Funding Statement**: This study did not receive any specific grants from funding agencies in the public, commercial, or not-for-profit sectors. MIURA CO., LTD. did not provide any research funding to IVF Nagata Clinic, its collaborative research partner, and each facility conducted the research using its own internal budgets. MIURA CO.,LTD. provided financial support in the form of salaries, research materials, and conference participation and travel expenses for authors [Emi Sugizaki, Shaimaa Ibrahim, Yuki Hashiguchi, Genki Koyama, Yasuo Nakamura, and Yuji Haishima], however, it has no additional role in study design, data collection and analysis, decision to publish, or preparation of the manuscript. The specific roles of these authors are described in the Author Contributions section.

**Competing Interests Statement**: This study did not receive any specific funding other than financial support in the form of salaries, research materials, and conference participation and travel from the authors’ affiliated institutions. No author received any compensation or honorarium for consulting, speaking engagements, presentations, speaker bureau services, writing, educational events, or expert testimony. MIURA CO., LTD. manufactures and sells ozone/hydroxy peroxide mixed gas sterilizer and the information has been disclosed in the Materials and methods section of the manuscript and on the company website: https://www.miuraz.co.jp/product/industry/xz.html.

Yuji Haishima has involved as a co-inventor of the patent ‘P2016-154835A’ titled ‘Decontamination apparatus and method’, and Emi Sugizaki, Shaimaa Ibrahim, Genki Koyama, Yasuo Nakamura, and Yuji Haishima are also co-inventors of the pending patent ‘P2025-71735A, titled ‘Removal method of hydrogen peroxide’, both of which are related intellectual properties to this study. The patent information has also been disclosed on the following websites: https://www.j-platpat.inpit.go.jp/p0200 and https://www.j-platpat.inpit.go.jp/p0200, respectively.

None of the authors serve on a Data Safety Monitoring Board or Advisory Board. Yuji Haishima serves as a council member of the Japanese Society for Biomaterials without compensation. None of the authors or their family members hold shares or stock options. No authors received equipment, supplies, pharmaceuticals, medical writing assistance, gifts, or other services from external sources. No authors have other financial or non-financial interests. These disclosures do not alter our adherence to PLOS ONE policies on sharing data and materials.

**Academic editor: Journal requirement 4**

We note that you have included the phrase “data not shown” in your manuscript. Unfortunately, this does not meet our data sharing requirements. PLOS does not permit references to inaccessible data. We require that authors provide all relevant data within the paper, Supporting Information files, or in an acceptable, public repository. Please add a citation to support this phrase or upload the data that corresponds with these findings to a stable repository (such as Figshare or Dryad) and provide and URLs, DOIs, or accession numbers that may be used to access these data. Or, if the data are not a core part of the research being presented in your study, we ask that you remove the phrase that refers to these data.

【Reply】 Addition and modification

Based on this comment, protocols and results of static contact angle, tensile strength, and FT-IR analyses were added to the ‘Materials and methods” and “Results” sections of the revised manuscript. As part of this revision, Table 4 and a new Fig 1 were added, and the original figures were renumbered accordingly. The sentence containing the phrase ‘(data not shown)’ was also revised in the ‘Discussion’ section. For details of the revised text, please refer to Sections 1, 2/2), 2/4) to 2/6), and 3/3) in the attached “List of Changes”.

**Academic editor: Journal requirement 5**

If the reviewer comments include a recommendation to cite specific previously published works, please review and evaluate these publications to determine whether they are relevant and should be cited. There is no requirement to cite these works unless the editor has indicated otherwise.

【Reply】 Not applicable

With my kindest regards and best wishes,

Yuji Haishima, Ph.D.

Innovative Decontamination Technology Development and Evaluation Center

MIURA CO.,LTD.

List of change

All additions and modifications have been highlighted in red in the revised manuscript. Deleted words and text are marked with a double strikethrough. The page and line numbers listed here correspond to those in the revised manuscript with track changes.

1. Materials and methods: Addition (page 8, line180 to page 9, line 190)

A new subsection titled ‘Physicochemical analysis’ was added as follows; Physicochemical analyses were performed according to previously reported methods [16]. Briefly, Fourier transform infrared (FT-IR) spectra and static contact angles of the soft polyvinyl chloride (sPVC), silicone (SI), polyamide (PA), and polysulfone (PSU) sheets (10 x 50 x t2 mm: NISHIYMA Corp., Tokyo, Japan) were measured using an FT/IR-4600 instrument (JASCO Corporation, Tokyo, Japan) and a B100 model attached to an LDH-100 automated dispenser (ASUMI-GIKEN, Tokyo, Japan), respectively. The tensile strengths of these materials (JIS K7139, A2, t4 mm) were analyzed using an Autograph AG-X with MWG-20kN as a load cell (SHIMADZU CORPORATION, Kyoto, Japan) at a tensile speed of 50 mm/min. The properties of each sample were measured before and after ozone/hydrogen peroxide mixed gas sterilization in ET mode, in which the default number of pulses (six) was increased to nine to provide 1.5 times the normal load.

2. Results

1) Page 13, lines 267 and 268: Modification

The header was modified from ‘Removal performance of residual hydrogen peroxide by drying treatment’ to ‘Removal of residual hydrogen peroxide’.

2) Page 13, line 284 to page 14, line 302: Addition

A new subsection titled ‘Material compatibility to ozone/hydrogen peroxide mixed gas treatment’ was added as follows: The effects of ozone/hydrogen peroxide mixed gas sterilization in ET mode on sPVC, SI, PA, and PSU, which are the component materials used in intrauterine insemination and embryo transfer catheters, were evaluated. As shown in Fig 1, there were no significant changes in the FT-IR spectra of these materials before and after treatment. As shown in Table 4, the static contact angles of the intact materials were 95.0 ± 3.2° for sPVC, 113 ± 1.8° for SI, 78.3 ± 2.0° for PA, and 95.7 ± 4.1° for PSU. The treatment did not significantly affect these values, which were 91.2 ± 1.9°, 112 ± 1.8°, 84.0 ± 1.7°, and 87.1 ± 1.7°, respectively. In addition, each tensile strength parameter was similar before and after treatment (Table 4), indicating that these materials were not affected by the sterilization.

Table 4 and a new Fig 1 were also added to this section.

3) Page 14, line 304: Modification

The header was modified from ‘Effect on mice and human embryo development’ to ‘Effect on mouse and human embryo development’.

4) Page 15, lines 311 and 315: Modification

With addition of new Fig 1, original Fig 1 was renumbered to Fig 2.

5) Page 15, lines 318, 322, and 324

With addition of new Fig 1, original Fig 2 was renumbered to Fig 3.

6) Page 16, lines 333 and 342

With addition of new Fig 1, original Fig 3 was renumbered to Fig 4.

3. Discussion

1) Page 17, lines 369 to 375: Addition

Information useful for readers to understand the characteristics of HCPT was added as follows: Werner et al. demonstrated clear differences in sensitivity between the endotoxin test and HCPT in a comparative study using intraocular lenses artificially contaminated with Gram-negative or Gram-positive bacteria. In that study, HCPT detected pyrogens in all samples, whereas all samples were negative in the endotoxin test [32]. The responsiveness to pyrogens in HCPT containing MAT varies depending on the conditions of the blood donors or the cell lots. Therefore, the responsiveness is corrected using IL-6/standard endotoxin and absorbance/standard endotoxin calibration curves generated for each test, enabling accurate quantification of pyrogens.

2) Page 18, line 386: Modification

Original reference no.32 was renumbered to no.33.

3) Page 18, lines 392 to 397: Modification

The phrase ‘data not shown’ was deleted and the material name ‘nylon’ was corrected to ‘polyamide’ because Nylon was originally trademark, as follows: In addition, ozone/hydrogen peroxide gas sterilization does not affect material properties such as Fourier Transform Infrared Spectroscopy spectra, static contact angle, and tensile strength of not only polypropylene and polystyrene [16], which correspond to raw materials for cell culture utensils, but also soft polyvinyl chloride, silicone, polyamide, and polysulfone, which comprise intrauterine insemination and embryo transfer catheters.

4) Page 18, line 401: Modification

Original reference nos.33 and 34 were renumbered to nos.34 and 35, respectively.

5) Page 18, line 401 to page 19, line 418: Addition and modification

The discussion was reconstructed by citing several references that evaluated the effects of hydrogen peroxide on germ cells, and related original discussion of the results obtained in this study was also revised as follows: Although hydrogen peroxide has been recognized as one of the most toxic oxidizing species for human spermatozoa, it plays a dual role that acts as a mediator of normal sperm function at low concentrations and exhibits cytotoxicity at high concentrations [36]. Mararajah et al. reported that sperm parameters changed when samples collected from 32 healthy, fertile men were exposed to 200 μM (6.80 µg/mL) of hydroxy peroxide [37]. Nguyen et al. reported that hydroxy peroxide at a concentration of 3 mM (102 µg/mL) caused DNA damage and completely inhibited sperm motility in mice, as well as delays and arrest in embryonic development [38]. On the other hand, it has been reported that incubation of spermatozoa with low concentrations (25 µM, 0.85 µg/mL) of hydrogen peroxide stimulates the process of capacitation; this stimulation is maintained not only through the early appearance of hyperactivated motility and the ability to undergo acrosome reaction in response to the calcium ionophore A23187, but also through an increased rate of sperm-oocyte fusion [39,40]. In this study, although the concentration of residual hydrogen peroxide in the culture medium was estimated to be 0.37 ± 0.03 µg/mL when embryos were cultured in the µDrop GPS dish in test group B, the blastocyst formation rate and sperm motility were reduced. These results suggest that germ cells may be adversely affected even at such low concentrations of hydrogen peroxide.　However, by applying drying treatment at 80°C for 24 h after sterilization to reduce the concentration to 0.14 ± 0.02 µg/mL, the effects on human sperm culture and embryo development were effectively eliminated.

6) Page 19, lines 418 to 424: Deletion

With the revision described in Section 5/5), the related original discussion was deleted.

4. Conclusions (Page 20, lines 438 to 444: Deletion and modification)

The ‘Conclusion’ section except for the last sentence, was revised as follows: Commercially available disposable products such as cell culture equipment and catheters used for IVF are not necessarily pyrogen-free, and some products contain non-negligible amounts of pyrogens. However, the present study suggests that pathogen-free manufacturing of these products, with no adverse effects on germ cells, is now possible using ozone/hydrogen peroxide gas sterilization and subsequent drying technologies.

5. References

1) Page 25, lines 549 to 551 : Addition

A new reference was added as no.32.

2) Page 25, lines 552, 555, and 558: Modification

Original reference nos.32 to 34 were renumbered to Nos.33 to 35.

3) Page 25, line 561 to page 26, line 575: Addition

New references were added as nos. 36 to 40.
