# Supplementary material for: Quality assessment of endotoxin contamination in consumables used for assisted reproductive technology
Source: PLoS One. 2026 Jun 17;21(6):e0341246. doi: 10.1371/journal.pone.0341246 (PMC13274850; doi:10.1371/journal.pone.0341246)
Supplement: Peer review history

# Peer review: Decision Letter 0

**DOI:** 10.1371/journal.pone.0341246.r001

**Authors:** Mohammad Faezi Ghasemi

3 May 2026

PONE-D-25-68905

Quality assessment of endotoxin contamination in consumables used for assisted reproductive technology

PLOS One

Dear Dr. Haishima,

Thank you for submitting your manuscript to PLOS ONE. After careful consideration, we feel that it has merit but does not fully meet PLOS ONE’s publication criteria as it currently stands. Therefore, we invite you to submit a revised version of the manuscript that addresses the points raised during the review process.

==============================

**Dear respected Authors,**

**Based on the evaluation from our reviewers, I have decided that your manuscript requires a major revision. Please follow the reviewers' recommendations point by point and submit your revised version to PLOS One.**

**Best regards,**

==============================

Please submit your revised manuscript by Jun 17 2026 11:59PM. If you will need more time than this to complete your revisions, please reply to this message or contact the journal office at plosone@plos.org. When you're ready to submit your revision, log on to https://www.editorialmanager.com/pone/ and select the 'Submissions Needing Revision' folder to locate your manuscript file.

Please include the following items when submitting your revised manuscript:

- A letter that responds to each point raised by the academic editor and reviewer(s). You should upload this letter as a separate file labeled 'Response to Reviewers'.
- A marked-up copy of your manuscript that highlights changes made to the original version. You should upload this as a separate file labeled 'Revised Manuscript with Track Changes'.
- An unmarked version of your revised paper without tracked changes. You should upload this as a separate file labeled 'Manuscript'.

If you would like to make changes to your financial disclosure, please include your updated statement in your cover letter. Guidelines for resubmitting your figure files are available below the reviewer comments at the end of this letter.

If applicable, we recommend that you deposit your laboratory protocols in protocols.io to enhance the reproducibility of your results. Protocols.io assigns your protocol its own identifier (DOI) so that it can be cited independently in the future. For instructions see: https://journals.plos.org/plosone/s/submission-guidelines#loc-laboratory-protocols. Additionally, PLOS ONE offers an option for publishing peer-reviewed Lab Protocol articles, which describe protocols hosted on protocols.io. Read more information on sharing protocols at https://plos.org/protocols?utm_medium=editorial-email&utm_source=authorletters&utm_campaign=protocols.

As the corresponding author, your ORCID iD is verified in the submission system and will appear in the published article. PLOS supports the use of ORCID, and we encourage all coauthors to register for an ORCID iD and use it as well. Please encourage your coauthors to verify their ORCID iD within the submission system before final acceptance, as unverified ORCID iDs will not appear in the published article. *Only*  the individual author can complete the verification step; PLOS staff *cannot*  verify ORCID iDs on behalf of authors.

We look forward to receiving your revised manuscript.

Kind regards,

Mohammad Faezi Ghasemi, Ph.D

Academic Editor

PLOS One

Journal Requirements:

When submitting your revision, we need you to address these additional requirements.

1. Please ensure that your manuscript meets PLOS ONE's style requirements, including those for file naming. The PLOS ONE style templates can be found at

https://journals.plos.org/plosone/s/file?id=wjVg/PLOSOne_formatting_sample_main_body.pdf and

https://journals.plos.org/plosone/s/file?id=ba62/PLOSOne_formatting_sample_title_authors_affiliations.pdf

2. We note that your Data Availability Statement is currently as follows: “All relevant data are within the manuscript and its Supporting Information files.”

Please confirm at this time whether or not your submission contains all raw data required to replicate the results of your study. Authors must share the “minimal data set” for their submission. PLOS defines the minimal data set to consist of the data required to replicate all study findings reported in the article, as well as related metadata and methods (https://journals.plos.org/plosone/s/data-availability#loc-minimal-data-set-definition).

For example, authors should submit the following data:

- The values behind the means, standard deviations and other measures reported;

- The values used to build graphs;

- The points extracted from images for analysis.

Authors do not need to submit their entire data set if only a portion of the data was used in the reported study.

If your submission does not contain these data, please either upload them as Supporting Information files or deposit them to a stable, public repository and provide us with the relevant URLs, DOIs, or accession numbers. For a list of recommended repositories, please see https://journals.plos.org/plosone/s/recommended-repositories.

If there are ethical or legal restrictions on sharing a de-identified data set, please explain them in detail (e.g., data contain potentially sensitive information, data are owned by a third-party organization, etc.) and who has imposed them (e.g., an ethics committee). Please also provide contact information for a data access committee, ethics committee, or other institutional body to which data requests may be sent. If data are owned by a third party, please indicate how others may request data access.

3. Thank you for stating the following in the Financial Disclosure section:

“The author(s) received no specific funding for this work.”

We note that one or more of the authors are employed by a commercial company: “Innovative Decontamination Technology Development and Evaluation Center, MIURA CO. LTD., Yokohama-shi, Kanagawa, Japan” and “Low-Temperature Sterilizer Technical Section, Medical Hospital Equipment Technical Department, MIURA CO. LTD., Matsuyama-shi, Ehime, Japan”

1. Please provide an amended Funding Statement declaring this commercial affiliation, as well as a statement regarding the Role of Funders in your study. If the funding organization did not play a role in the study design, data collection and analysis, decision to publish, or preparation of the manuscript and only provided financial support in the form of authors' salaries and/or research materials, please review your statements relating to the author contributions, and ensure you have specifically and accurately indicated the role(s) that these authors had in your study. You can update author roles in the Author Contributions section of the online submission form.

Please also include the following statement within your amended Funding Statement.

“The funder provided support in the form of salaries for authors [insert relevant initials], but did not have any additional role in the study design, data collection and analysis, decision to publish, or preparation of the manuscript. The specific roles of these authors are articulated in the ‘author contributions’ section.”

If your commercial affiliation did play a role in your study, please state and explain this role within your updated Funding Statement.

2. Please also provide an updated Competing Interests Statement declaring this commercial affiliation along with any other relevant declarations relating to employment, consultancy, patents, products in development, or marketed products, etc.

Within your Competing Interests Statement, please confirm that this commercial affiliation does not alter your adherence to all PLOS ONE policies on sharing data and materials by including the following statement: "This does not alter our adherence to  PLOS ONE policies on sharing data and materials.” (as detailed online in our guide for authors http://journals.plos.org/plosone/s/competing-interests) . If this adherence statement is not accurate and  there are restrictions on sharing of data and/or materials, please state these. Please note that we cannot proceed with consideration of your article until this information has been declared.

Please include both an updated Funding Statement and Competing Interests Statement in your cover letter. We will change the online submission form on your behalf.

4. We note that you have included the phrase “data not shown” in your manuscript. Unfortunately, this does not meet our data sharing requirements. PLOS does not permit references to inaccessible data. We require that authors provide all relevant data within the paper, Supporting Information files, or in an acceptable, public repository. Please add a citation to support this phrase or upload the data that corresponds with these findings to a stable repository (such as Figshare or Dryad) and provide and URLs, DOIs, or accession numbers that may be used to access these data. Or, if the data are not a core part of the research being presented in your study, we ask that you remove the phrase that refers to these data.

5. If the reviewer comments include a recommendation to cite specific previously published works, please review and evaluate these publications to determine whether they are relevant and should be cited. There is no requirement to cite these works unless the editor has indicated otherwise.

[Note: HTML markup is below. Please do not edit.]

Reviewers' comments:

Reviewer's Responses to Questions

**Comments to the Author**

1. Is the manuscript technically sound, and do the data support the conclusions?

The manuscript must describe a technically sound piece of scientific research with data that supports the conclusions. Experiments must have been conducted rigorously, with appropriate controls, replication, and sample sizes. The conclusions must be drawn appropriately based on the data presented.

Reviewer #1: Partly

Reviewer #2: Yes

**********

2. Has the statistical analysis been performed appropriately and rigorously?

Reviewer #1: Yes

Reviewer #2: Yes

**********

3. Have the authors made all data underlying the findings in their manuscript fully available?

The PLOS Data policy requires authors to make all data underlying the findings described in their manuscript fully available without restriction, with rare exception (please refer to the Data Availability Statement in the manuscript PDF file). The data should be provided as part of the manuscript or its supporting information, or deposited to a public repository. For example, in addition to summary statistics, the data points behind means, medians and variance measures should be available. If there are restrictions on publicly sharing data—e.g. participant privacy or use of data from a third party—those must be specified.

Reviewer #1: Yes

Reviewer #2: Yes

**********

4. Is the manuscript presented in an intelligible fashion and written in standard English?

PLOS ONE does not copyedit accepted manuscripts, so the language in submitted articles must be clear, correct, and unambiguous. Any typographical or grammatical errors should be corrected at revision, so please note any specific errors here.

Reviewer #1: Yes

Reviewer #2: Yes

**********

5. Review Comments to the Author

Please use the space provided to explain your answers to the questions above. You may also include additional comments for the author, including concerns about dual publication, research ethics, or publication ethics. (Please upload your review as an attachment if it exceeds 20,000 characters)

Reviewer #1: The study suggests that contaminated containers “may reduce ART success rates,” but no clinical outcome data are provided to support this claim. Extrapolating from laboratory results (embryo growth and sperm motility) of potential biological effects to actual clinical outcomes of ART is not conclusive. Therefore, conclusions about reduced ART success rates should be interpreted in the discussion section appropriately, with a review of previous research, or supported by clinical outcome data

In this study, the observed concentrations of hydrogen peroxide were compared with the reported toxicity threshold for human mesenchymal stem cells. However, mesenchymal stem cells are not biologically equivalent to human gametes or embryos, which are generally known to be more sensitive to oxidative stress. Therefore, using MSC data as a reference for reproductive cell safety may not be appropriate. This section is discussed in detail in the Discussion.

The human cell-based pyrogen test (HCPT) plays a key role in the conclusions of this study; more details on the validation and robustness of this method would be helpful.

Were spike and recovery tests performed for the specific substances tested?

Was inter-donor variability assessed?

How many independent blood donors were included in each test condition?

Reviewer #2: This work is relevant and sould be published. Small linguistic issues, however: Headers: "Effect on mice and human embryo development" should be "Effect on mouse and human embryo development" ; "Removal performance of residual hydrogen peroxide by drying treatment" should be "Removal of residual hydrogen peroxide" .

**********

6. PLOS authors have the option to publish the peer review history of their article (what does this mean?). If published, this will include your full peer review and any attached files.

If you choose “no”, your identity will remain anonymous but your review may still be made public.

**Do you want your identity to be public for this peer review?**  For information about this choice, including consent withdrawal, please see our Privacy Policy.

Reviewer #1: **Yes:** Ashraf Kariminik

Reviewer #2: No

**********

[NOTE: If reviewer comments were submitted as an attachment file, they will be attached to this email and accessible via the submission site. Please log into your account, locate the manuscript record, and check for the action link "View Attachments". If this link does not appear, there are no attachment files.]

To ensure your figures meet our technical requirements, please review our figure guidelines: https://journals.plos.org/plosone/s/figures

You may also use PLOS’s free figure tool, NAAS, to help you prepare publication quality figures: https://journals.plos.org/plosone/s/figures#loc-tools-for-figure-preparation.

NAAS will assess whether your figures meet our technical requirements by comparing each figure against our figure specifications.

# Author comment: Author response to Decision Letter 1

**DOI:** 10.1371/journal.pone.0341246.r002

21 May 2026

Reply to comments of reviewers and journal requirements

Manuscript number: PONE-D-25-68905

Title: Quality assessment of endotoxin contamination in consumables used for assisted reproductive technology

Authors: Hiroyuki Tomari, et al.

We sincerely appreciate your comprehensive and insightful review of our manuscript. Your constructive comments have been invaluable in helping us improve the quality and clarity of our work. We have responded to your comments below and made the necessary revisions accordingly. All corrections are listed in the attached “List of Changes”.

Reviewer 1: comment 1

The study suggests that contaminated containers “may reduce ART success rates,” but no clinical outcome data are provided to support this claim. Extrapolating from laboratory results (embryo growth and sperm motility) of potential biological effects to actual clinical outcomes of ART is not conclusive. Therefore, conclusions about reduced ART success rates should be interpreted in the discussion section appropriately, with a review of previous research, or supported by clinical outcome data.

【Reply】 Deletion and modification

We completely agree with this comment. Since the impact of pyrogen contamination levels identified by HCPT on ART success rates is discussed in the “Discussion” section (the revised manuscript; page 18, lines 381 to 387) with citations to the relevant literature reported to date, we deleted the first sentence and modified the second sentence in the 'Conclusion' section as they were not appropriate for the conclusion of our manuscript. For details of the revised text, please refer to Section 4 in the attached “List of Changes”.

Reviewer 1: comment 2

In this study, the observed concentrations of hydrogen peroxide were compared with the reported toxicity threshold for human mesenchymal stem cells. However, mesenchymal stem cells are not biologically equivalent to human gametes or embryos, which are generally known to be more sensitive to oxidative stress. Therefore, using MSC data as a reference for reproductive cell safety may not be appropriate. This section is discussed in detail in the Discussion.

【Reply】 Deletion, modification, and addition

According to this comment, the discussion comparing the effects of hydrogen peroxide on hMSCs and germ cells was deleted and reconstructed by using several references evaluating the effects of hydrogen peroxide on germ cells. The discussion of the results obtained in this study was also revised to ensure consistency with the context. With this revision, new references 36-40 were added. For details of the revised text, please refer to Sections 3/5), 3/6), and 5/1) in the attached “List of Changes”.

Reviewer 1: comment 3

The human cell-based pyrogen test (HCPT) plays a key role in the conclusions of this study; more details on the validation and robustness of this method would be helpful.

【Reply】 Addition

Although the characteristics of HCPT and the status of its standardization in the United States, Europe, and Japan are originally summarized in the ‘Discussion’ section (revised manuscript; page 17, lines 369 to 375), further information useful to readers was added in response to Comments 3-5 from Reviewer 1. With the revision, new reference 32 was added, and the original references after no.32 were renumbered. For details of the revised text, please refer to Sections 3/1) and 5 in the attached “List of Changes”.

Reviewer 1: comment 4

Were spike and recovery tests performed for the specific substances tested?

【Reply】 Addition

Although spike-and-recovery tests of the cell culture utensils and medical devices used in this study were not performed, such tests have been conducted in our laboratory and other research teams using many products consisting of plastics and metals. Compared with standard endotoxin tests using water-for-injection extracts as samples, the direct HCPT offers up to 2,000-fold, 80-fold, and 800-fold higher sensitivity for detecting pyrogens from metal, plastic, and rubber materials, respectively.

The difference in recovery ratios between the HCPT and endotoxin tests are already mentioned in the 'Introduction' and 'Discussion' sections. However, based on the comments 3 and 4, new reference 32 was added to the “Discussion” section as a representative example of a recovery test (see reply to comment 3).

Reviewer 1: comment 5

Was inter-donor variability assessed? How many independent blood donors were included in each test condition?

【Reply】 Addition

Two blood donors participated in this study, and HCPT was performed several times on different dates. As pointed out in this comment, responsiveness to pyrogens in the HCPT varies not only among donor but also according to the blood collection date, even the same donor. Therefore, responsiveness was corrected using IL-6/standard endotoxin and absorbance/standard endotoxin calibration curves generated for each test, enabling accurate quantification of pyrogens. This information was added to the ‘Discussion’ section based on comments 3 and 5 described above (see reply to comment 3).

Reviewer 2: comment 1

This work is relevant and should be published. Small linguistic issues, however: Headers: "Effect on mice and human embryo development" should be "Effect on mouse and human embryo development"; "Removal performance of residual hydrogen peroxide by drying treatment" should be "Removal of residual hydrogen peroxide".

【Reply】 Modification

The headers in the ‘Results’ section were modified according to this comment.

Academic editor: Journal requirement 1

Please ensure that your manuscript meets PLOS ONE's style requirements, including those for file naming. The PLOS ONE style templates can be found at https://journals.plos.org/plosone/s/file?id= wjVg/ PLOSOne_formatting_sample_main_body.pdf and https://journals.plos.org/plosone/s/file?id= ba62/ PLOSOne _ formatting_sample_title_authors_affiliations.pdf.

【Reply】 It has been confirmed before submission of the revised version.

Academic editor: Journal requirement 2

We note that your Data Availability Statement is currently as follows: “All relevant data are within the manuscript and its Supporting Information files.”

Please confirm at this time whether or not your submission contains all raw data required to replicate the results of your study. Authors must share the “minimal data set” for their submission. PLOS defines the minimal data set to consist of the data required to replicate all study findings reported in the article, as well as related metadata and methods (https://journals.plos.org/plosone/s/data-availability#loc-minimal-data-set-definition).

For example, authors should submit the following data:

- The values behind the means, standard deviations and other measures reported;

- The values used to build graphs;

- The points extracted from images for analysis.

Authors do not need to submit their entire data set if only a portion of the data was used in the reported study.

If your submission does not contain these data, please either upload them as Supporting Information files or deposit them to a stable, public repository and provide us with the relevant URLs, DOIs, or accession numbers. For a list of recommended repositories, please see https://journals.plos.org/ plosone/s/recommended-repositories.

If there are ethical or legal restrictions on sharing a de-identified data set, please explain them in detail (e.g., data contain potentially sensitive information, data are owned by a third-party organization, etc.) and who has imposed them (e.g., an ethics committee). Please also provide contact information for a data access committee, ethics committee, or other institutional body to which data requests may be sent. If data are owned by a third party, please indicate how others may request data access.

【Reply】 No Changes

All relevant data are included in the manuscript.

Academic editor: Journal requirement 3

Thank you for stating the following in the Financial Disclosure section: “The author(s) received no specific funding for this work.” We note that one or more of the authors are employed by a commercial company: “Innovative Decontamination Technology Development and Evaluation Center, MIURA CO. LTD., Yokohama-shi, Kanagawa, Japan” and “Low-Temperature Sterilizer Technical Section, Medical Hospital Equipment Technical Department, MIURA CO. LTD., Matsuyama-shi, Ehime, Japan”

1. Please provide an amended Funding Statement declaring this commercial affiliation, as well as a statement regarding the Role of Funders in your study. If the funding organization did not play a role in the study design, data collection and analysis, decision to publish, or preparation of the manuscript and only provided financial support in the form of authors' salaries and/or research materials, please review your statements relating to the author contributions, and ensure you have specifically and accurately indicated the role(s) that these authors had in your study. You can update author roles in the Author Contributions section of the online submission form.

Please also include the following statement within your amended Funding Statement. “The funder provided support in the form of salaries for authors [insert relevant initials], but did not have any additional role in the study design, data collection and analysis, decision to publish, or preparation of the manuscript. The specific roles of these authors are articulated in the ‘author contributions’ section.” If your commercial affiliation did play a role in your study, please state and explain this role within your updated Funding Statement.

2. Please also provide an updated Competing Interests Statement declaring this commercial affiliation along with any other relevant declarations relating to employment, consultancy, patents, products in development, or marketed products, etc. Within your Competing Interests Statement, please confirm that this commercial affiliation does not alter your adherence to all PLOS ONE policies on sharing data and materials by including the following statement: "This does not alter our adherence to PLOS ONE policies on sharing data and materials.” (as detailed online in our guide for authors http://journals.plos.org/ plosone/s/competing-interests) . If this adherence statement is not accurate and there are restrictions on sharing of data and/or materials, please state these. Please note that we cannot proceed with consideration of your article until this information has been declared.

Please include both an updated Funding Statement and Competing Interests Statement in your cover letter. We will change the online submission form on your behalf.

【Reply】 Modification

We understand that if any of the authors are affiliated with commercial entities, it will be subject to rigorous scrutiny regarding potential conflicts of interest. For this reason, not only for PLOS One but for all journal submissions, we have provided the ICMJE Disclosure Form for all authors as ‘Supplementary Information’ to assist the Chief Editor in making a decision.

This study did not receive any specific grant from funding agencies in the public, commercial, or not-for-profit sectors. MIURA CO.,LTD. has not provided any research funding to IVF Nagata Clinic, a collaborative research partner, and each facilities conducted the research using its own in-house budgets. Including patent application status related to this study, all information are disclosed in the ICMJE Disclosure Forms. In addition, regardless of the type of organization, fair salaries and in-house budgets are generally not subject to disclosure under the Transparency Guidelines. From these reasons, we previously inquired via email about the necessity of this requirement, but we have not received a response to date.

Since we have never been asked to provide such additional statements from scientific journal offices in past, we understood this to be a specific editorial policy of PLOS One. Although the content seems to be the same and just expressed differently, the Funding Statement and Competing Interests Statement have been updated as follows and also included in the cover letter submitted with our revised manuscript, as requested.

Funding Statement: This study did not receive any specific grants from funding agencies in the public, commercial, or not-for-profit sectors. MIURA CO., LTD. did not provide any research funding to IVF Nagata Clinic, its collaborative research partner, and each facility conducted the research using its own internal budgets. MIURA CO.,LTD. provided financial support in the form of salaries, research materials, and conference participation and travel expenses for authors [Emi Sugizaki, Shaimaa Ibrahim, Yuki Hashiguchi, Genki Koyama, Yasuo Nakamura, and Yuji Haishima], however, it has no additional role in study design, data collection and analysis, decision to publish, or preparation of the manuscript. The specific roles of these authors are described in the Author Contributions section.

Competing Interests Statement: This study did not receive any specific funding other than financial support in the form of salaries, research materials, and conference participation and travel from the authors’ affiliated institutions. No author received any compensation or honorarium for consulting, speaking engagements, presentations, speaker bureau services, writing, educational events, or expert testimony. MIURA CO., LTD. manufactures and sells ozone/hydroxy peroxide mixed gas sterilizer and the information has been disclosed in the Materials and methods section of the manuscript and on the company website: https://www.miuraz.co.jp/product/industry/xz.html.

Yuji Haishima has involved as a co-inventor of the patent ‘P2016-154835A’ titled ‘Decontamination apparatus and method’, and Emi Sugizaki, Shaimaa Ibrahim, Genki Koyama, Yasuo Nakamura, and Yuji Haishima are also co-inventors of the pending patent ‘P2025-71735A, titled ‘Removal method of hydrogen peroxide’, both of which are related intellectual properties to this study. The patent information has also been disclosed on the following websites: https://www.j-platpat.inpit.go.jp/p0200 and https://www.j-platpat.inpit.go.jp/p0200, respectively.

None of the authors serve on a Data Safety Monitoring Board or Advisory Board. Yuji Haishima serves as a council member of the Japanese Society for Biomaterials without compensation. None of the authors or their family members hold shares or stock options. No authors received equipment, supplies, pharmaceuticals, medical writing assistance, gifts, or other services from external sources. No authors have other financial or non-financial interests. These disclosures do not alter our adherence to PLOS ONE policies on sharing data and materials.

Academic editor: Journal requirement 4

We note that you have included the phrase “data not shown” in your manuscript. Unfortunately, this does not meet our data sharing requirements. PLOS does not permit references to inaccessible data. We require that authors provide all relevant data within the paper, Supporting Information files, or in an acceptable, public repository. Please add a citation to support this phrase or upload the data that corresponds with these findings to a stable repository (such as Figshare or Dryad) and provide and URLs, DOIs, or accession numbers that may be used to access these data. Or, if the data are not a core part of the research being presented in your study, we ask that you remove the phrase that refers to these data.

【Reply】 Addition and modification

Based on this comment, protocols and results of static contact angle, tensile strength, and FT-IR analyses were added to the ‘Materials and methods” and “Results” sections of the revised manuscript. As part of this revision, Table 4 and a new Fig 1 were added, and the original figures were renumbered accordingly. The sentence containing the phrase ‘(data not shown)’ was also revised in the ‘Discussion’ section. For details of the revised text, please refer to Sections 1, 2/2), 2/4) to 2/6), and 3/3) in the attached “List of Changes”.

Academic editor: Journal requirement 5

If the reviewer comments include a recommendation to cite specific previousl

Attachment Submitted filename: Response to Reviewers.docx

# Peer review: Decision Letter 1

**DOI:** 10.1371/journal.pone.0341246.r003

**Authors:** Mohammad Faezi Ghasemi, Mohammad Faezi Ghasemi

4 Jun 2026

Quality assessment of endotoxin contamination in consumables used for assisted reproductive technology

PONE-D-25-68905R1

Dear Dr. Haishima,

We’re pleased to inform you that your manuscript has been judged scientifically suitable for publication and will be formally accepted for publication once it meets all outstanding technical requirements.

Within one week, you’ll receive an e-mail detailing the required amendments. When these have been addressed, you’ll receive a formal acceptance letter and your manuscript will be scheduled for publication.

An invoice will be generated when your article is formally accepted. Please note, if your institution has a publishing partnership with PLOS and your article meets the relevant criteria, all or part of your publication costs will be covered. Please make sure your user information is up-to-date by logging into Editorial Manager at Editorial Manager® and clicking the ‘Update My Information' link at the top of the page. For questions related to billing, please contact billing support.

If your institution or institutions have a press office, please notify them about your upcoming paper to help maximize its impact. If they’ll be preparing press materials, please inform our press team as soon as possible -- no later than 48 hours after receiving the formal acceptance. Your manuscript will remain under strict press embargo until 2 pm Eastern Time on the date of publication. For more information, please contact onepress@plos.org.

Kind regards,

Mohammad Faezi Ghasemi, Ph.D

Academic Editor

PLOS One

Additional Editor Comments (optional):

Reviewers' comments:

Reviewer's Responses to Questions

**Comments to the Author**

1. If the authors have adequately addressed your comments raised in a previous round of review and you feel that this manuscript is now acceptable for publication, you may indicate that here to bypass the “Comments to the Author” section, enter your conflict of interest statement in the “Confidential to Editor” section, and submit your "Accept" recommendation.

Reviewer #1: All comments have been addressed

Reviewer #2: All comments have been addressed

**********

2. Is the manuscript technically sound, and do the data support the conclusions?

The manuscript must describe a technically sound piece of scientific research with data that supports the conclusions. Experiments must have been conducted rigorously, with appropriate controls, replication, and sample sizes. The conclusions must be drawn appropriately based on the data presented.

Reviewer #1: Yes

Reviewer #2: Yes

**********

3. Has the statistical analysis been performed appropriately and rigorously?

Reviewer #1: Yes

Reviewer #2: Yes

**********

4. Have the authors made all data underlying the findings in their manuscript fully available?

The PLOS Data policy requires authors to make all data underlying the findings described in their manuscript fully available without restriction, with rare exception (please refer to the Data Availability Statement in the manuscript PDF file). The data should be provided as part of the manuscript or its supporting information, or deposited to a public repository. For example, in addition to summary statistics, the data points behind means, medians and variance measures should be available. If there are restrictions on publicly sharing data—e.g. participant privacy or use of data from a third party—those must be specified.

Reviewer #1: Yes

Reviewer #2: Yes

**********

5. Is the manuscript presented in an intelligible fashion and written in standard English?

PLOS ONE does not copyedit accepted manuscripts, so the language in submitted articles must be clear, correct, and unambiguous. Any typographical or grammatical errors should be corrected at revision, so please note any specific errors here.

Reviewer #1: Yes

Reviewer #2: Yes

**********

6. Review Comments to the Author

Please use the space provided to explain your answers to the questions above. You may also include additional comments for the author, including concerns about dual publication, research ethics, or publication ethics. (Please upload your review as an attachment if it exceeds 20,000 characters)

Reviewer #1: The authors have addressed all of my previous comments thoroughly. The methods, results, discussion, and conclusion sections have been appropriately revised based on the feedback provided. I have no further major concerns regarding the scientific content or presentation of this manuscript. The authors have responded professionally and the manuscript appears to be in compliance with standard ethical guidelines.

Reviewer #2: Appropriate changes have been made. The authors have successfully met all concerns raised by the reviewers.

**********

7. PLOS authors have the option to publish the peer review history of their article (what does this mean?). If published, this will include your full peer review and any attached files.

If you choose “no”, your identity will remain anonymous but your review may still be made public.

**Do you want your identity to be public for this peer review?**  For information about this choice, including consent withdrawal, please see our Privacy Policy.

Reviewer #1: **Yes:** Ashraf Kariminik

Reviewer #2: No

**********

# Editor report: Acceptance letter

**DOI:** 10.1371/journal.pone.0341246.r004

**Authors:** Mohammad Faezi Ghasemi, Mohammad Faezi Ghasemi

PONE-D-25-68905R1

PLOS One

Dear Dr. Haishima,

I'm pleased to inform you that your manuscript has been deemed suitable for publication in PLOS One. Congratulations! Your manuscript is now being handed over to our production team.

At this stage, our production department will prepare your paper for publication. This includes ensuring the following:

* All references, tables, and figures are properly cited

* All relevant supporting information is included in the manuscript submission,

* There are no issues that prevent the paper from being properly typeset

You will receive further instructions from the production team, including instructions on how to review your proof when it is ready. Please keep in mind that we are working through a large volume of accepted articles, so please give us a few days to review your paper and let you know the next and final steps.

Lastly, if your institution or institutions have a press office, please let them know about your upcoming paper now to help maximize its impact. If they'll be preparing press materials, please inform our press team within the next 48 hours. Your manuscript will remain under strict press embargo until 2 pm Eastern Time on the date of publication. For more information, please contact onepress@plos.org.

You will receive an invoice from PLOS for your publication fee after your manuscript has reached the completed accept phase. If you receive an email requesting payment before acceptance or for any other service, this may be a phishing scheme. Learn how to identify phishing emails and protect your accounts at https://explore.plos.org/phishing.

If we can help with anything else, please email us at customercare@plos.org.

Thank you for submitting your work to PLOS ONE and supporting open access.

Kind regards,

PLOS ONE Editorial Office Staff

on behalf of

Dr. Mohammad Faezi Ghasemi

Academic Editor

PLOS One
